# Better Late Than Never: Predictors of Delayed COVID-19 Vaccine Uptake in Poland

**DOI:** 10.3390/vaccines10040528

**Published:** 2022-03-29

**Authors:** Marcin Piotr Walkowiak, Jan Domaradzki, Dariusz Walkowiak

**Affiliations:** 1 Department of Preventive Medicine, Poznan University of Medical Sciences, 60-781 Poznań, Poland; marcinwalkowiak@ump.edu.pl; 2 Department of Social Sciences and Humanities, Poznan University of Medical Sciences, 60-806 Poznań, Poland; jandomar@ump.edu.pl; 3 Department of Organisation and Management in Health Care, Poznan University of Medical Sciences, 60-356 Poznań, Poland

**Keywords:** COVID-19, vaccination, vaccination coverage, trust in vaccine, interventions to increase vaccination coverage, social capital, public health, vaccine hesitancy

## Abstract

In this study, regression models were created to explain the increase of COVID-19 vaccination rates in 378 Polish sub-regions. In order to trace the factors that could explain the willingness to delay vaccination, vaccination rates were compared for age groups of 20 years and more for 30 June 2020 and 31 January 2021. Initially high vaccination rates, rather than leading to the gradual exhaustion of the pool of those wishing to get vaccinated, were a very good predictor of the share of the remainder willing to do so, which increased the divergence between sub-regions in nominal vaccination rates. Support for Eurosceptic and anti-establishment parties was a strong predictor of persistent vaccine hesitancy. Ideological divergence from the mainstream appeared to reinforce vaccine hesitancy, and this relationship remained highly relevant even when controlling for possible time or spatial lag. Markers of social inclusion and social capital—voter turnout and employment rate—remained statistically significant even when controlling for time lag, thus implying clear relevance of trust in the public message. The share of the population with higher education remained a highly relevant factor as well, though in the 20–39 age bracket it predicted a higher vaccination rate, while in all older brackets it was a negative predictor—this implies that those people had already made up their minds. Delaying vaccination seems predominantly explainable by political views, as well as social exclusion and the historical specificity of sub-regions. On a regional level, there was actually a paradoxical Spearmans Rho correlation (0.641) between the share of population refusing mandatory vaccination for kids and the percentage of people receiving a COVID-19 vaccine, which further undermines the idea that overall observed vaccine hesitancy was in any meaningful way affected by anti-vaccine movements.

## 1. Introduction

The National Vaccination Programme in Poland was launched on 27 December 2020, and later expanded to include adolescents aged over 16 and children above 12 years old (May and June 2021, respectively). Until 14 February 2022, the first dose was received by 22,449,048 of Poles, while 19,308,786 received the second one, which means that 21,998,598 (50.34%) are fully vaccinated. Thus, only 58.53% of the Polish population received at least one dose [1].

It is difficult to consider vaccination programmes as successful in any of the post-communist countries [2]. Moreover, even compared to its neighbours, Poland does not do well [3]. Various reasons for this were indicated, but there is no certainty that this phenomenon can be considered explained [4,5,6]. Even before the vaccination campaign started, the individualistic approach of Polish citizens and their unwillingness to join the activities for the common good in Poland were indicated [7].

Various measures have been taken all over the world to raise the level of vaccinations in society. The course of the pandemic changed the way it was perceived; as a consequence, the attitude of citizens to vaccination has changed as well. In Japan, Harada and Watanabe [8] found that vaccine acceptance rates increased from 40.6% to 85.5% in five months in 2021. Le Maréchal et al. [9] established that among those vaccinated in May and June 2021, almost two-thirds were sure they would not have preferred to receive the vaccine as early as January. In the UK, Chaudhuri et al. [10] found “that trust in public sector officials play a key factor in the low vaccination rates particularly seen in at-risk groups”. In a comparative analysis of Japan, Israel and Hungary, Goodwin et al. [11] established that vaccine willingness was greatest among those who trusted their government. Recent American results [12] show decreasing vaccine delay and refusal. Purvis et al. [13] documented trusted sources of vaccine information among hesitant adopters. Kennedy et al. [14] reported that “more temporally distant the availability of COVID vaccines, the more likely individuals were to want to get the shot immediately”. Results from France, by Bajos et al. [15], indicate that “people at the bottom of the social hierarchy, in terms of level of education or financial resources, were more likely to refuse the COVID-19 vaccine”. In the Netherlands, Yousuf et al. [16] found lower household annual income, migrant background and lower educational level as predictors of vaccine hesitancy.

The low level of COVID-19 immunisation was not the only problem in Poland; it turned out that regional differences in individual regions amounted to hundreds. Apart from regions with almost full vaccine coverage, there were also those where only one-in-ten people were vaccinated [5]. Regional differences are not surprising in many countries; what is surprising is the magnitude of differences. Moreover, apart from an ill-designed and clearly unsuccessful vaccination campaign, the vaccination itself has become the subject of a political game. The supporters of the ruling party were allegedly unvaccinated, and therefore their political opponents accused the government of not paying due attention to vaccination. Recent opinion polls have shown that there is a difference in the acceptance of COVID-19 vaccinations between supporters of various parties [4]. These results should be surprising for all sides of the political dispute in Poland. As it was established, education and population density were positively related to low vaccine hesitancy [5]. As it turned out, however, correlations between markers of social exclusion and vaccination are negative. Moreover, the study showed that people who have not yet been vaccinated and declare that they want to do so give evasive responses, explaining their decisions by the lack of time or the desire to vaccinate later. Of course, on a rational level, such responses were to be expected, although the results of the study do not in any way bring us closer to the answers who the people who have not been vaccinated yet are. The real reasons for vaccine delay are not clear, but we also do not know how the vaccine-hesitant can be persuaded to be vaccinated. In contrast to other countries of the region, Poland did not implement COVID-19 passports or any other effective incentive to vaccinate [2]. Thus, an increase in the vaccination rate cannot be explained in general by compulsion, but by people genuinely and freely deciding to get vaccinated.

The purpose of this study is to research vaccine hesitancy, where this term is not merely a euphemism of vaccine rejection, but refers to citizens literally being “hesitant” and needing more time to decide. The ability to identify this group is crucial for future vaccination campaigns, as these people are neither enthusiasts nor hard line opponents, but exactly those who should be a target of any campaign, as otherwise they may vaccinate regrettably late. Receiving honest answers in any questionnaire would be challenging, as respondents would be likely to have to admit postponing, being indecisive or having changed their mind, which would lead to answers including a significant amount of rationalisation of their past decisions. Instead, a more promising route for analysing the problem seems to be a statistical analysis of actual behaviour, and an attempt to identify those who might become the target of subsequent campaigns.

## 2. Methods

Information on the percentage of vaccinated population in particular regions was taken from the Polish Ministry of Health [1]. The data were collected as of 30 July 2021 and 31 January 2022. Subdivision of data for age groups was determined by data availability, which was provided for the following age groups: 12–19, 20–39, 40–59, 60–69 and 70–79. For the purpose of this study, group 12–19 has been omitted; first, this group is highly heterogeneous, as it includes both minors under parental care and young adults making their own decisions, thus it is likely to be governed by somewhat diverging factors; and second, vaccination of the 12–15 age group started on 7 June 2021, so this group would have had less than a month to receive the first dose of vaccine before being prematurely classified as late adopters. People who received at least a single dose are considered vaccinated. This methodological decision was partially caused by data availability, but also simplified our initial assumptions.

This study analyses, on the basis of an earlier study assessing socio-economic factors affecting the initial willingness to vaccinate, the actual factors contributing to delayed vaccine uptake [5]: the results and the voter turnout of 2020 presidential election in Poland, as well as—additionally—the results of the previous 2015 one (of which only those relating to one of the presidential candidates were eventually included). The Polish political scene is dynamic, and therefore the 2020 candidates were not the same group as five years earlier. In order to achieve comparability, the same key explanatory variables as in the original study [4] were used, including higher education, employment rate and COVID-19 deaths. The political variables were derived from the National Electoral Commission website [16]. Voter turnout in the second round of the presidential election of 2020 was used as a proxy indicator of social engagement.

Prior studies showed that ideological views tend to be a stronger predictor of vaccine hesitancy than demographics [5,17,18,19]. The relation is especially clear in relation to worldviews that included conspiratorial beliefs, hierarchical worldview, contrarianism and individualism. In consequence, in order to find voters who were statistically more likely to fit any element of this description, right-wing and polarising candidates from recent elections were selected.

In the second round of presidential election, the country was split into two camps. On one side was the ruling right-wing party Law and Justice (EU parliament faction: European Conservatives and Reformists), whose narrative rests on soft Euroscepticism, economic interventionism and social conservatism. It enjoys the greatest support among older, religious people who highly value conservatism and patriotism. In addition, it is supported by the working class, union members and people with a secondary education. On the other side there were typical liberals who had shifted towards the centre: the Civic Platform (EU parliament faction: European People’s Party), supported mainly by younger voters, those better off, university-educated, living in the western part of the country or in big cities. In that particular election, the Civic Platform, due to its secularism and pro-EU stance, also received the votes of a relatively small left-wing segment of electorates.

We used the votes for incumbent Andrzej Duda, the candidate of the Law and Justice party in 2020, as a proxy for moderate support of anti-establishment right-wing. The vote in the first round of presidential election is the only moment when voters can reveal their true preference without much potential for strategic voting, and therefore we used the initial vote in 2020 for Krzysztof Bosak, a candidate of Confederation Liberty and Independence (former EU faction: Europe of Freedom and Direct Democracy, currently non-affiliated) to gauge support for the individualistic and non-mainstream right-wing. It should be noted that Confederation Liberty and Independence was the only political force that opposed pandemic laws and restrictions, while within its ranks it allows heterogeneous viewpoints ranging from classical liberalism to the far-right. The vote for Paweł Kukiz, the leader of protest movement Kukiz’15, in the first round of presidential election of 2015, represents for us general anti-establishment dissatisfaction that is hard to place on the right-left axis. Within this movement there were a few members of parliament who were openly anti-vaccine, though this fact should not be overestimated, as they were a minority even within their own ranks.

The only available source of data on the percentage of people with higher education and of those in employment in each sub-region (*powiat*) was the 2011 census. While those data are clearly old, in the previous study they had high predictive power. Due to territorial changes in that period, however, the sample had to be narrowed from 380 to 378 sub-regions.

The number of COVID-19 deaths per 10 000 in sub-regions was measured by four variables, which were a combination of two factors. The first factor was whether death was officially classified as solely caused by COVID-19, or listed as due also to additional conditions. The second factor was whether the death has happened until 30th June 2021, or between 1st July 2021 and 30th January 2022. The data we used were from the official data of the Polish Ministry of Health [20].

Any factor related directly to this phenomenon was gauged through population density, which was taken from Statistics Poland [21]; this was because cities had higher vaccination rates.

As some social phenomena are measured with some proxy indicators that are highly correlated, there is a risk that in a set models the same phenomenon would be represented by a different variable, which would prevent direct comparison. Thus, in order to avoid this, a variable, to be included, would have to end up as statistically significant in at least 3 out of 12 models. Otherwise, it would be dropped and model would be recalculated without it.

All variables in regression models, except constant ones, had to be statistically significant with *p* < 0.05 in order to be included. Statistical analysis was performed in Gretl 2019d, except for spatial regression, which was calculated in GeoDa 1.18.0, the program also used to create maps.

## 3. Results

**Figure 1:**
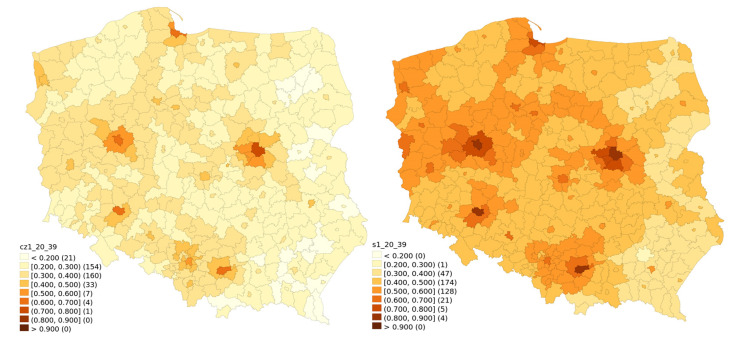
Vaccination rate for the 20–39 age bracket for 30 June 2021 and 31 January 2022.

**Figure 2:**
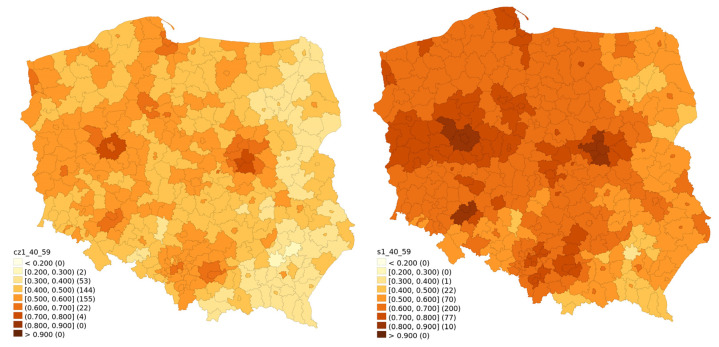
Vaccination rate for the 40–59 age bracket for 30 June 2021 and 31 January 2022.

**Figure 3:**
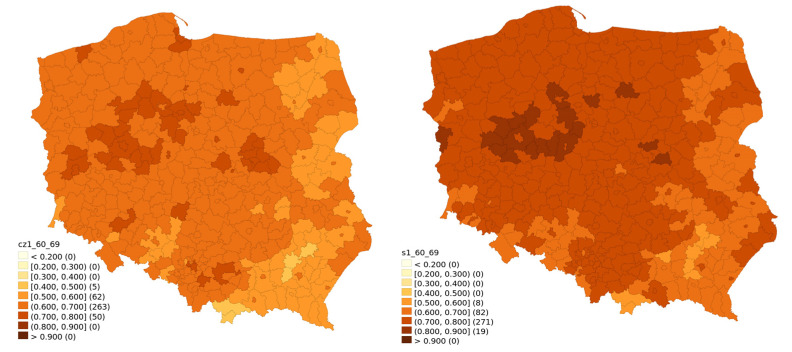
Vaccination rate for the 60–69 age bracket for 30 June 2021 and 31 January 2022.

**Figure 4:**
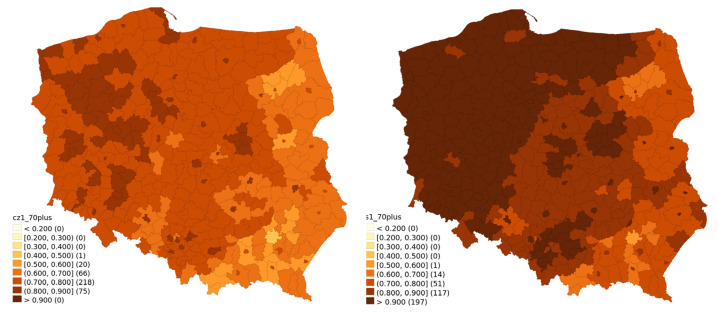
Vaccination rate for the 70 and above age bracket for 30 June 2021 and 31 January 2022.

Temporal changes of vaccination rates are visualised for different age classes between 30 June 2020 and 31 January. They are presented in Figure 1 (ages 20–39), Figure 2 (ages 40–59), Figure 3 (ages 60–69) and Figure 4 (ages 70 and above). Some patterns, such as higher vaccination rates in cities and lowest near the eastern border of Poland, seem to apply regardless of the time of observation and of the age group.

The highest nominal increase in the percentage of those vaccinated was observed in the 20–39 age group (Figure 1). This is not surprising, considering that the people from this group had the shortest window of opportunity for getting vaccinated. For this youngest group, the historical divide between the east and the west is somewhat less visible, while the difference between big cities with suburbs and the rest of the country is more pronounced.

Figure 2 presents the 40–59 age bracket, which can be described as an intermediary group, in which there also exist the tendencies generally observed in other age groups, such as the regional divide and modest growth, albeit in a slightly weaker form.

Figure 3 shows that the age bracket 60–69 had actually the lowest nominal increase. This age group was old enough to be characterised by relatively high initial vaccination rate, while paradoxically young enough to not generally require much assistance in reaching a vaccination point.

The models that explain the factors affecting the later adoption of COVID-19 vaccine among age groups are presented in the following tables: Table 1—age bracket 20–39; Table 2—age bracket 40–59; Table 3—age bracket 60–69; and Table 4—age bracket 70 and above. For each age group, there are three models: without any lag; with time lag; and with spatial lag. For all brackets and variants, there is clearly a potential for including a lag, which for all models, except one, could account for approximately half (43% to 70%) of the observed variability.

There is a clear general trend regarding the impact of political variables, with votes for all the three analysed party candidates negatively related to subsequent vaccination. Wherever statistically significant, each individual vote for Law and Justice candidate had the weakest impact, though this party had by a degree of magnitude more votes than the other analysed ones. Votes for Kukiz had a moderately negative relationship with vaccination. Votes for Confederation had the strongest impact—in 3 out of 4 models without lag, they had a much higher impact than one would expect even if none of their voters were vaccinated. For the 60–69 age group in time lagged model, the impact of voting for the Confederation changed sign. This fact, while unusual on its own, is actually consistent with all other time lagged models, where the impact of those votes was significantly reduced, as well for all time lagged models.

There was at least one variable in each model that was a marker of social participation (i.e., voter turnout, employment rate, or both). While for younger age brackets both markers were possible, for the oldest age bracket the only relevant one was voter turnout, which was logical, as old age pensioners should be less affected by the job market. In all cases, regardless of other variables and time or spatial lag, those two variables retained a positive relation with vaccination rate.

In almost all age groups, the number of sole COVID-19 deaths until 30th June 2020 retained its positive impact on subsequent vaccination, and even on the further growth of vaccination rates. This variable lost its significance in time lagged models, though there this initial phenomenon should be covered by time lag. The impact of higher education was only positive in the youngest age bracket without time lag, while negative in all the remaining cases.

Our models, even before including increase from taking into account lag variables, had quite a high determination factor of 74.6% for the 20–39 age bracket, 57.4% for those aged 40–59, 39.2% for the 60–69 group and 64.1% for those aged 70 and above. The relatively low determination factor in the age group 60–69 was initially caused by the highest vaccination rate, and thus subsequent increases were relatively modest and less affected by general trends.

## 4. Discussion

Our models clearly show that the initial vaccination pattern is a good predictor of subsequent vaccine uptake among the remaining population. However, the role of particular factors was clearly changing. Political views, especially support for parties willing to directly challenge the establishment and the prevailing narrative, become a good predictor of vaccine hesitancy. These findings are in line with the research from various countries [17,18,19,22,23,24]. While it was possible to fit subsequent vaccine uptake with population density as an extra statistically significant variable, the methodology required to narrow down the number of variables to only those that would be statistically significant in more models, to avoid drawing far-reaching conclusions from something that is most likely a statistical fluke.

The number of initial deaths classified as exclusively caused by COVID-19 remained a statistically significant factor in 3 out of 4 age groups, which makes it hard to dismiss it as some purely statistical anomaly, even though it seems to measure some less obvious mechanism. It clearly does not measure COVID-19 deaths in general, as in that period actual COVID-19 deaths were seriously undercounted [25]. It was unlikely to even measure actual deaths that were indeed solely caused by COVID-19, as on the sub-region level it was negatively correlated with deaths caused by COVID-19 combined with other underlying causes. This variable does not work either when calculated for a longer period, but this fact can be actually easily explained when we realise that any subtle impact of this variable may have been overshadowed by the subsequent high vaccination rate, leading to a reduction of deaths. Such long-term impact of this variable is somewhat ambiguous to interpret. It could either mean that those deaths were still taken directly into consideration, and convinced a tiny, but statistically significant number of people to vaccinate. Alternatively, it could also mean an indirect impact, where this factor led to an initial vaccination boost, while later it led to a further increase through social proof. Additionally, the data on the daily number of first-time vaccinations show an increase after the relatively peaceful summer of 2021 ended and the fifth wave started taking its toll. This pattern implies which was the key argument to convince the vaccine-hesitant. Contoli et al. [26] established the influence of having had a death from COVID-19 among family or friends on the willingness of the elderly to get vaccinated. Such a relationship was also confirmed by Salali and Uysal [27]. However, Singh et al. [28] found higher exposure to information about deaths and other serious conditions caused by the COVID-19 vaccination to be associated with a lower vaccine uptake.

The role of higher education as a predictor was not uniform. It increased the vaccination rate in the lowest age bracket, while decreasing it in all older groups. This result is not so surprising, as initially, education was generally a strong factor explaining the willingness to vaccinate early, so particularly in well-vaccinated age brackets, those shunning vaccination were more likely to lack education. In the youngest age group, however, there was less time to vaccinate and the general vaccination rate was lower, so higher education remained a factor increasing the vaccination rate.

Our models and data visualisation clearly show a very strong geographic pattern in vaccination rates. For the youngest age bracket, however, there was a slightly bigger role of big cities and their proximity, while for the oldest demographic the geographic pattern matched closer Poland’s historical borders and mass population movements.

The observed vaccine hesitancy cannot be blamed on the vocal, growing, but in reality small anti-vaccine movement. First, studies gauging general opinion show very high and effectively uniform acceptance for child vaccination in Poland [29], which highly contrasts with attitudes towards COVID-19 vaccines. An actual refusal of obligatory vaccination is relatively rare, and applies to less than one per cent of cases. Moreover, when analysed on a provincial level, the relation goes in the opposite direction—Spearman’s Rho between obligatory vaccine refusal and getting vaccinated against COVID-19 in all age groups of 20 or more is 0.641 (*p* = 0.009), and even when narrowed to the 20–39 age bracket it remains 0.606 (*p* = 0.015). This would rather imply that the main choice is not between vaccine hesitancy and vaccine acceptance, but between vaccinations being some default, immutable, obligatory treatment for children, and vaccination as elective treatment to embrace or refuse, based on individual opinion and changing situation.

In all 12 models, a variable representing social cohesion and trust (i.e., employment rate or voter turnover) remained statistically significant, and in all 12 cases retained the same sign. As this variable retained significance in spatial lag, it can not be dismissed as some purely regional specificity, while retaining significance even in time lagged models clearly rules out that it was just merely social pressure. It is the most likely to have been some proxy indicator for social capital; the willingness to trust the official advice. Thus, it seems that apart from socio-demographic and economic determinants of vaccine hesitancy in Poland identified in previous studies [4,5,6], the low level of intake of COVID-19 vaccination in the country can be also explained by a generally low level of social capital. Indeed, research shows that active membership in various social organisations and participation in elections in post-communist countries is one of the lowest in Europe [30,31]. Although in the past thirty years, Poland succeeded in terms of political and economic transformation, on the social level it has created a huge “sociological vacuum”; prosocial attitudes, so crucial for developing social capital, have not been shaped. Consequently, many individuals do not feel attached to their community, are socially dysfunctional and feel connected to their families rather than to local communities [32]. If any feeble signs to the contrary can at all be noted, they seem limited to the group of young adults, some of whom were active during the first waves of the COVID-19 pandemic [33,34].

Sociological research suggests that although Polish society is characterised by a strong sense of solidarity and the willingness to help others, such engagement in various charity activities is often episodic and does not last long, whereas a long-term engagement is often delegated to formal organisations, including the state. Moreover, over the years, opinion polls showed that Polish society is overcome by a general mistrust toward others, including public institutions, as less than 22% of Poles believe that most people can be trusted. Moreover, while Poles mainly trust their family members, co-workers and neighbours, social trust in such public institutions as political parties, government, public administration and courts is much lower than in other European countries. Thus, because the basic unit that forges social bonds between its members is the family, one may agree with Lewenstein who suggests that the Polish society is a “family society” [35]. Thus, although the level of trust increases with education and income, in terms of generalised trust Poland ranks very low among the EU countries [32,36,37]. This, in turn, makes it impossible for Poles to undertake effective collective actions or engage in them. Lack of trust towards their own representatives was so great that in all age groups in models without lag, the percentage of people who voted for the sitting president was a statistically significant negative predictor of a subsequent increase in vaccination rate. As the president was engaged in pro-vaccination campaigns, it was leading to a situation in which people did not trust the official message, even when expressed by a person they themselves elected a year earlier.

There are many reasons for this, as insights from sociology suggest. First, while for historical reasons civic society in Poland was always developing in opposition to the state, after the transformation intellectual and political elites were also focused on building a democratic state and neglected other aspects. Consequently, citizens neither participated in the creation of new institutions nor understood the goals and direction of reforms, and thus felt alienated from the elites. Second, for many segments of society, rapid economic and political transformation was a very traumatic experience, which resulted in enduring alienation and the weakening of social participation. Third, after the transformation, neoliberal doctrine and the ideology of (economic) individualism superseded the idea of civic society. Fourth, due to globalisation and modernisation, many individuals felt helpless in the face of global corporations and international, bureaucratic organisations. Fifth, as in the communist system, participation in elections and membership in the communist party or various associations were mandatory, election absenteeism and not being involved in social organisations were often seen as an act of resistance. Sixth, one can observe a slow decomposition and disintegration of Polish society, as with the slow fading away of the working class, trade unions also lost many of their power; additionally, rural associations fade away as Polish countryside slowly transforms. Seventh, a strong family and a strong state are considered to be more important than intermediary structures, such as informal associations. Finally, due to historical reasons, Poles have a strong sense of mistrust, and public activity is not seen as something positive [38,39,40].

Numerous studies demonstrated that social capital is essential for creating health supporting environments, and contributes to the community development approach within health promotion [41,42,43]. It is also associated with individual health behaviours and psychological well-being [44]. For example, Nieminen et al. [45,46] showed that social participation and networks, high levels of trust and reciprocity are associated with non-smoking, physical activity, adequate duration of sleep and daily consumption of vegetables. Similarly, Poortinga [47] found that social capital was associated with self-rated health, as well as with different health behaviours.

For historical and political reasons, a sense of social belonging in Poland is much lower than in Western countries. Consequently, there is a growing body of evidence suggesting that, especially in times of (health) crisis, including epidemics, which call for collective action and for socially responsible behaviour, social capital is significantly associated with the adoption of such protective behaviours as social distancing, face masking, hand disinfection and vaccination. For example, research conducted by Rönnerstranda [48,49] indicates that social capital had a positive impact on individuals’ intention to accept vaccination against the 2009 H1N1 pandemic in Sweden and the United States. Furthermore, Chuang at al. [50] showed social capital to be positively associated with people’s intention to wash their hands more frequently, wear a face mask and accept vaccinations during an influenza pandemic in Taiwan. In contrast, in relation to the Ebola epidemic, Blair et al. showed that low social capital affected Liberians’ low precautions against the epidemic, as well as distrust, compliance with control policies and preventative behaviours [51].

Similar observations were made in the case of the current COVID-19 pandemic, as it is argued that it was better handled in countries where social capital is high. For example, some studies showed that while social capital had an impact on the spread of the virus, it also affected the number of COVID-19 infections and deaths [52,53,54,55]. In particular, social capital helped reduce non-essential activities, such as mobility directed at retail and recreational activities, promoted vaccination and reduced vaccination hesitancy [56,57].

Specific groups of messages may be accepted by unvaccinated people and specific messages are potentially effective for some particular subgroups, as demonstrated by James et al. [58]. As we have shown, the levels of vaccination in Poland vary between demographics. There are also significant regional differences. The failure to address the message to specific recipients means that the message that vaccinations are valid and people should be vaccinated does not reach anyone, and those who could be convinced by such messages were vaccinated long ago. The ability to reach the right audience with one’s message is the key to success—provided that the recipient has been properly identified.

## 5. Conclusions

A few key patterns were observed in the analysed period concerning the late adoption of the COVID-19 vaccine. The initial success of the vaccination campaign in a particular region was a good predictor of the remaining share of population likely to make up their mind. This finding is highly useful for future vaccination campaigns, as regardless of the availability of other data and of the differences in a local political landscape, it would immediately help establish how the vaccination rate is actually going to diverge regionally. Political views, as expressed during elections, also remain a highly valid factor of vaccine hesitancy, even when a politician is actively taking part in pro-vaccination campaigns. Social capital clearly plays an essential role in convincing people to get vaccinated, which is especially important for those who were initially indecisive. Higher education has a mixed impact, as it seems to be the general factor affecting early vaccination, though in badly vaccinated populations it remains a predictor of higher subsequent vaccine uptake.

## Figures and Tables

**Table 1:** Percentage of remaining population in age group 20–39 that were vaccinated against COVID-19 between 30 June 2021 and 31 January 2022.

Variable	Model without Lag	Model with Time Lag	Model with Spatial Lag
Const	0.218 (0.0393) ***	0.241 (0.0290) ***	0.163 (0.0301) ***
Time lag		0.430 (0.0373) ***	
Spatial lag			0.547 (0.0494) ***
Vote for Law and Justice	−0.155 (0.0217) ***	−0.0510 (0.0210) *	−0.0762 (0.0201) ***
Vote for Confederation	−1.534 (0.207) ***	−0.964 (0.188) ***	−0.810 (0.185) ***
Vote for Kukiz	−0.491 (0.0772) ***	−0.541 (0.066) ***	−0.378 (0.0688) ***
Voter turnout	0.129 (0.0606) *		
COVID−19 only deaths until 2021-06-30	0.0146 (0.00504) **		0.0119 (0.00457) **
Higher education	0.164 (0.0591) **	−0.152 (0.0542) ***	0.290 (0.0443) ***
Employment rate	0.473 (0.0637) ***	0.177 (0.0532) ***	0.193 (0.0370) ***
Adjusted R^2^	56.5%	67.4%	66.1%

* *p* < 0.05, ** *p* < 0.01, *** *p* < 0.001, standard deviation in bracket.

**Table 2:** Percentage of remaining population in age group 40–59 that were vaccinated against COVID-19 between 30 June 2021 and 31 January 2022.

Variable	Model without Lag	Model with Time Lag	Model with Spatial Lag
Const	0.0734 (0.0490)	0.0156 (0.0307)	−0.0687 (0.0283) *
Time lag		0.661 (0.0367) ***	
Spatial lag			0.699 (0.0376) ***
Vote for Law and Justice	−0.198 (0.0271) ***		−0.0568 (0.0163) ***
Vote for Confederation	−2.096 (0.258) ***	−1.038 (0.212) ***	−0.896 (0.193) ***
Vote for Kukiz	−0.295 (0.0964) **	−0.429 (0.0729) ***	
Voter turnout	0.448 (0.0756) ***		0.207 (0.0408) ***
COVID-19 only deaths until 2021-06-30	0.0228 (0.00629) ***		0.00962 (0.00485) *
Higher education	−0.222 (0.0738) **	−0.294 (0.0532) ***	
Employment rate	0.568 (0.0795) ***	0.334 (0.0619) ***	
Adjusted R^2^	57.4%	71.8%	75.9%

* *p* < 0.05, ** *p* < 0.01, *** *p* < 0.001, standard deviation in bracket.

**Table 3:** Percentage of remaining population in age group 60–69 that were vaccinated against COVID-19 between 30 June 2021 and 31 January 2022.

Variable	Model without Lag	Model with Time Lag	Model with Spatial Lag
Const	0.376 (0.0415) ***	−0.00192 (0.0429)	0.112 (0.0241) ***
Time lag		0.682 (0.0459) ***	
Spatial lag			0.568 (0.0505) ***
Vote for Law and Justice	−0.0728 (0.0267) **		
Vote for Confederation	−0.750 (0.258) **	0.693 (0.203) ***	−0.466 (0.180) ***
Vote for Kukiz	−0.299 (0.0948) **	−0.391 (0.0739) ***	
Voter turnout		0.107 (0.0539) *	
COVID-19 only deaths until 2021-06-30			
Higher education	−0.772 (0.0625) ***	−0.844 (0.0523) ***	−0.687 (0.0459) ***
Employment rate	0.219 (0.0654) ***		0.216 (0.0412) ***
Adjusted R^2^	39.2%	60.5%	55.0%

* *p* < 0.05, ** *p* < 0.01, *** *p* < 0.001, standard deviation in bracket.

**Table 4:** Percentage of remaining population in age group 70 and over that were vaccinated against COVID-19 between 30 June 2021 and 31 January 2022.

Variable	Model without Lag	Model with Time Lag	Model With Spatial Lag
Const	0.796 (0.0949) ***	−1.072 (0.0803) ***	0.299 (0.0909) ***
Time lag		2.301 (0.0772) ***	
Spatial lag			0.541 (0.0489) ***
Vote for Law and Justice	−0.997 (0.0634) ***	−0.239 (0.0449) ***	−0.537 (0.0686) ***
Vote for Confederation	−3.483 (0.611) ***		−1.4776 (0.544) **
Vote for Kukiz			
Voter turnout	0.958 (0.153) ***	0.270 (0.0913) ***	0.475 (0.139) ***
COVID-19 only deaths until 2021-06-30	0.0581 (0.0159) ***		0.0327 (0.0141) *
Higher education	−0.499 (0.179) **	−0.755 (0.0990) ***	0.407 (0.163) **
Employment rate			
Adjusted R^2^	64.1%	87.9%	72.9%

* *p* < 0.05, ** *p* < 0.01, *** *p* < 0.001, standard deviation in bracket.

## Data Availability

https://www.bdl.stat.gov.pl/BDL/dane/podgrup/temat; https://www.gov.pl/web/szczepimysie/mapa-punktow-szczepien; https://www.gov.pl/web/szczepienia-gmin/sprawdz-poziom-wyszczepienia-mieszkancow-gmin; https://www.gov.pl/web/szczepimysie/raport-szczepien-przeciwko-covid-19; https://www.pkw.gov.pl (accessed on 18 January 2022).

## Abbreviations

COVID-19: the coronavirus disease 2019; European Union: EU.
